# Identification of dyes on fabric exposed to lake and ocean water using near-infrared excitation Raman spectroscopy

**DOI:** 10.1039/d5ay01973g

**Published:** 2026-02-12

**Authors:** Claire Sasaki, Shannon Bober, Aidan P. Holman, Dmitry Kurouski

**Affiliations:** Department of Biochemistry and Biophysics, Texas A&M University, College Station, Texas 77843, USA

## Abstract

Forensic analysis of dyes present on fabric can provide a wealth of information about the relationship between the suspect and a crime scene. Such analysis requires analytical approaches that provide confirmatory information about the chemical structure of the dyes. A growing body of evidence indicates that near-Infrared excitation Raman spectroscopy (NIeRS) can be used for non-invasive and non-destructive detection and identification of colored fabric. However, it remains unclear how environmental factors such as water exposure could alter the accuracy of dye identification by NIeRS. To answer this question, we exposed cotton fabric colored by blue and magenta dyes to ocean and lake waters for 14 weeks. During this time, fabric was analyzed biweekly using a handheld NIeRS spectrometer. Chemometrics was used to process and learn from the acquired NIeRS spectra. Our results indicate that both ocean and lake water cause substantial fading of dyes. NIeRS revealed that fading rates are greater for lake than ocean water. Such water-triggered fading substantially lowered accuracy of NIeRS-based dye identification. We observed a dye-dependent decrease in prediction accuracy from 100% at week 0 to 30–80% by week 6. Nevertheless, built spectroscopic libraries enabled ~98% accurate identification of both dyes on fabric. These results indicate that water exposure possesses a great risk to NIeRS-based identification of dyes on fabric. However, a combination of NIeRS and artificial intelligence allowed for overcoming these limitations.

## Introduction

Coloured fabric materials are commonly found at crime scenes.^1,2^ Consequently, forensic examination of such fabric can be used to establish a connection between a suspect and a crime scene. However, forensic analysis of fibre is typically limited to the microscopic inspection of fibre structure and shape.^3,4^ Next, pattern recognition is performed to identify the relevance of the fabric samples found at crime scene and a reference or library samples.^5,6^ Subjective nature of such pattern-recognition analysis triggered the need for robust and reliable analytical approaches that can be used to advance forensic analysis of fabric.

Fluorescence and Infrared microscopy and spectroscopy can provide some information about the chemical nature of the fabric and dyes present on it.^7,8^ However, spectroscopic fingerprints of the analysed material are dependent on both nature of the fabric and dyes present on it. This cross-interference drastically complicates robustness of fluorescent-based analysis of fabric. Liquid chromatography (LC) and LC-mass spectrometry can identify dyes present in fabric with high sensitivity and specificity.^3,9,10^ However, both techniques are destructive, making the evidence unusable for further testing or presentation in its original state^3,11–13^ Our group discovered that dyes present on fabric could be detected and identified using near-Infrared excitation Raman spectroscopy (NIeRS).^14^ In NIeRS, 830 nm continuous-wavelength laser is used to illuminate the sample surface, which minimizes the fluorescence background in the acquired Raman spectra. Sample illumination triggered two types of scattering–elastic and inelastic. Elastically scattered photons are eliminated using optical filters. Inelastically scattered photons are collected by the instrument. Shifts in the photon energy of such photons corresponds to certain vibrations of the molecules present in the sample.^15,16^ Consequently, an acquisition of such photons allows for the non-invasive and non-destructive analysis of chemical structure and composition of the sample of interest.^17–20^ Our group demonstrated that NIeRS could be used for highly accurate analysis of a large number of dyes present on cotton fabric.^21^ We also investigated possible interference of substances of biological and non-biological origin for such NIeRS-based identification of dyes.^22^ Our previous results indicated that the presence of urine and semen on coloured fabric caused very little, if any, spectroscopic changes. However, blood contamination of fabric substantially changed the spectroscopic fingerprint of the colorants.^22^ In the current study, we investigate the effect of water-driven fading of blue and magenta dyes on the coloured cotton fabric. We also aim to reveal the extent to which such fading affects the NIeRS-based identification of dyes within 14-week time frame of fabric exposure to ocean and lake waters.

## Results and discussion

**Fig. 1:**
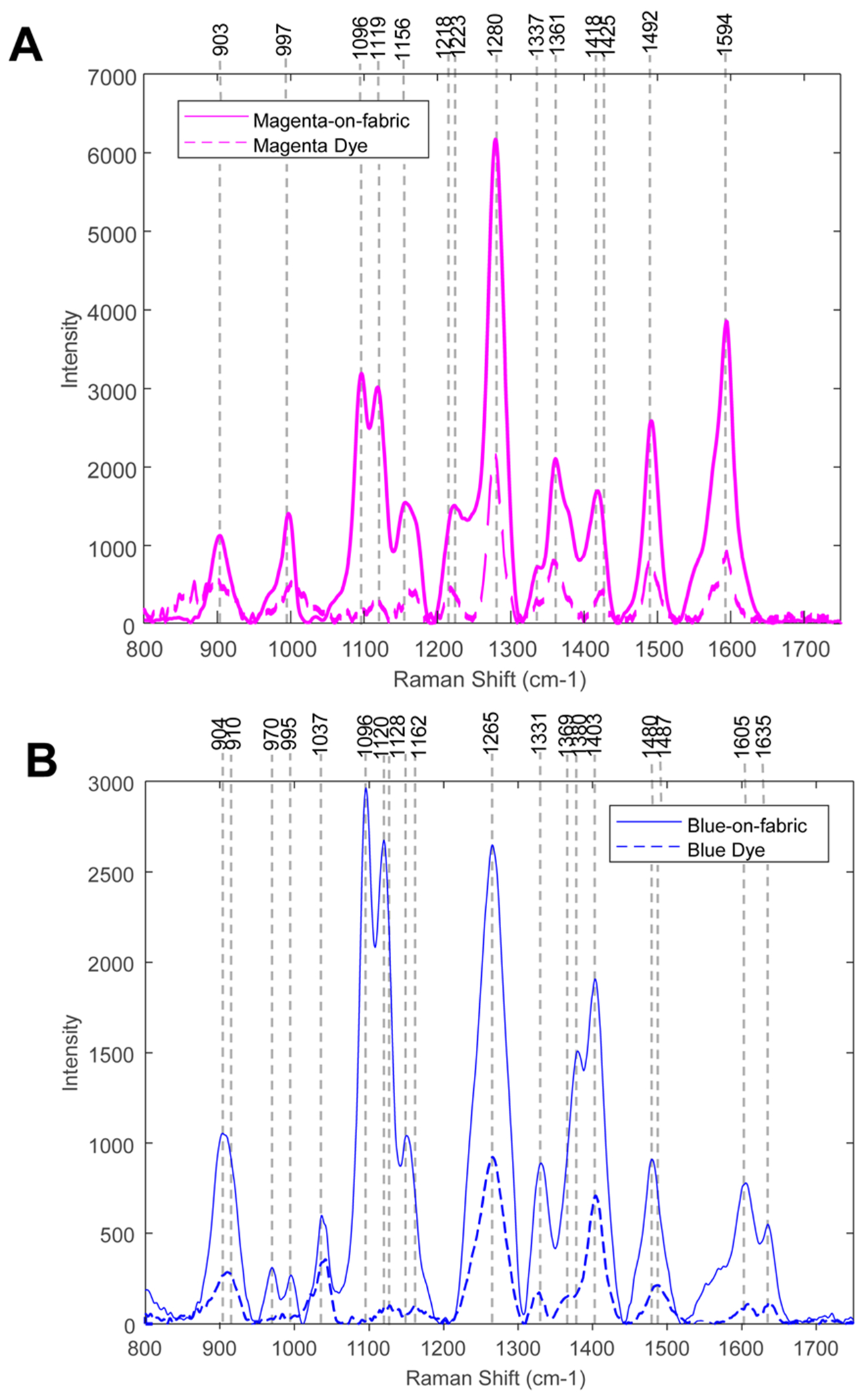
NIeRS spectra acquired from magenta-(A) and blue-(B) coloured fabric and dyes themselves.

First, we acquired NIeRS from coloured fabric and dyes themselves to ensure that NIeRS could detect the dyes present on fabric. Based on the observed vibrational bands in the corresponding pairs of spectra, we can conclude that NIeRS is capable of detecting both magenta and blue dyes present on fabric, Fig. 1 and Table 1. It should be noted that besides the vibrational bands that originated from the dyes, NIeRS was able to detect vibrational signatures of cotton fabric.^21,22^ Thus, in addition to the information about the dyes, NIeRS could be used to identify the chemical origin of fabric material.

**Fig. 2:**
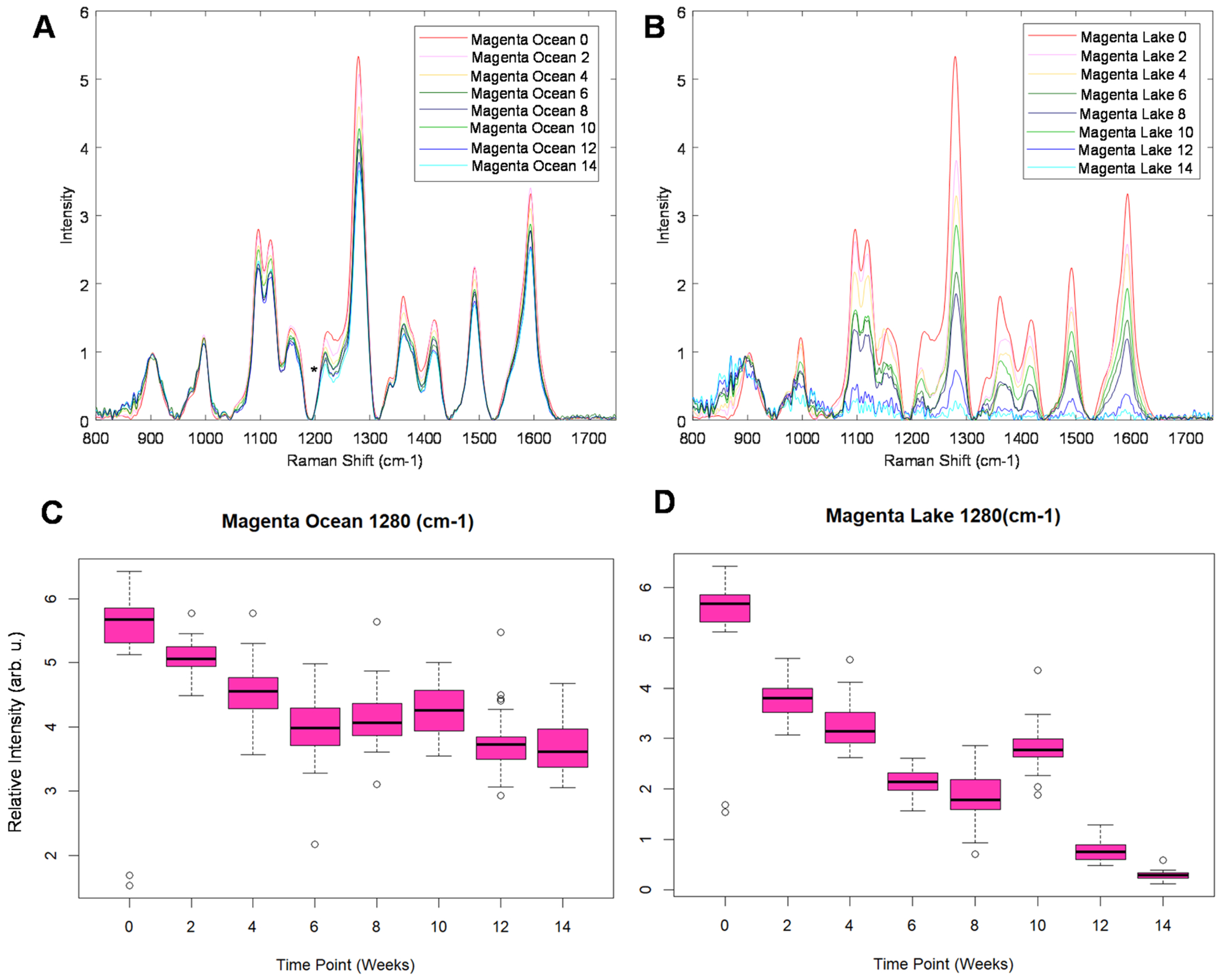
NIeRS spectra acquired from magenta-coloured cotton fabric exposed to ocean (A) and lake (B) waters. Corresponding changes in the 1280 cm^−1^ band are reflected in ANOVA graphs for the spectra acquired from ocean (C) and lake (D) water-exposed samples, as well as the control group (week 0).

Next, we acquired spectra from magenta-coloured fabric exposed to ocean and lake water, Fig. 2A and B. We observed a gradual decrease in the intensity of all vibrational bands as the duration of the sample exposure increased. Interestingly, lake water caused much stronger dye fading compared to lake water. This conclusion could be made based on ANOVA of 1280 cm^−1^ band, Fig. 2C, D, Tables S1 and S2. We observed a significantly smaller decrease in the intensity of this band in the spectra acquired from magenta dye exposed to ocean water for 2–14 weeks compared to lake water for the corresponding period of time. It should be noted that at week 14, lake water completely erased magenta dye present on the cotton fabric. It is important to point out that at this time point, not only spectroscopic signatures of the dye vanished, but also the vibrational fingerprints of cellulose, the major components of a cotton fabric. These results indicate that lake water caused degradation of cotton material which can explain the degradation of the dyes present on it.

**Fig. 3:**
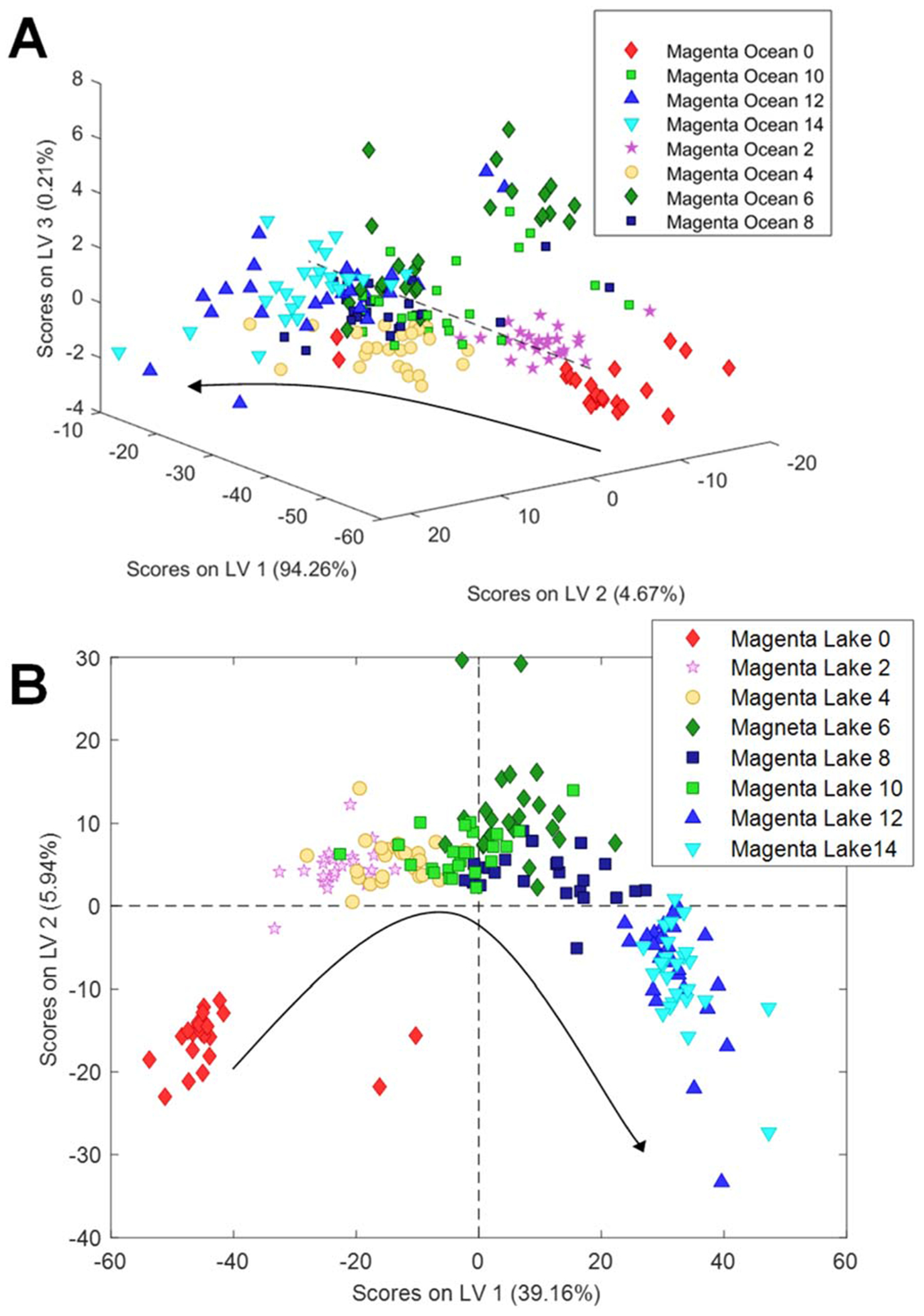
3D (A) and 2D (B) LV plots of NIeRS spectra acquired from magenta-coloured fabric exposed to ocean (A) and lake (B) waters for 2–14 weeks, as well as the control group (week 0). Cross-Validation (CV) Error and Root Mean Square Error of Cross-Validation (RMSECV) Plots for these PLS-DA models are shown in Fig. S1 and S2.

Chemometric analysis of the acquired spectra demonstrated clear trends in spectroscopic changes between spectral groups that represent different durations of water exposure (weeks 2–14), as well as the control group (week 0), Fig. 3. These spectroscopic changes enabled highly accurate identification of the duration of fabric exposure to both ocean and lake waters (discussed below).

**Fig. 4:**
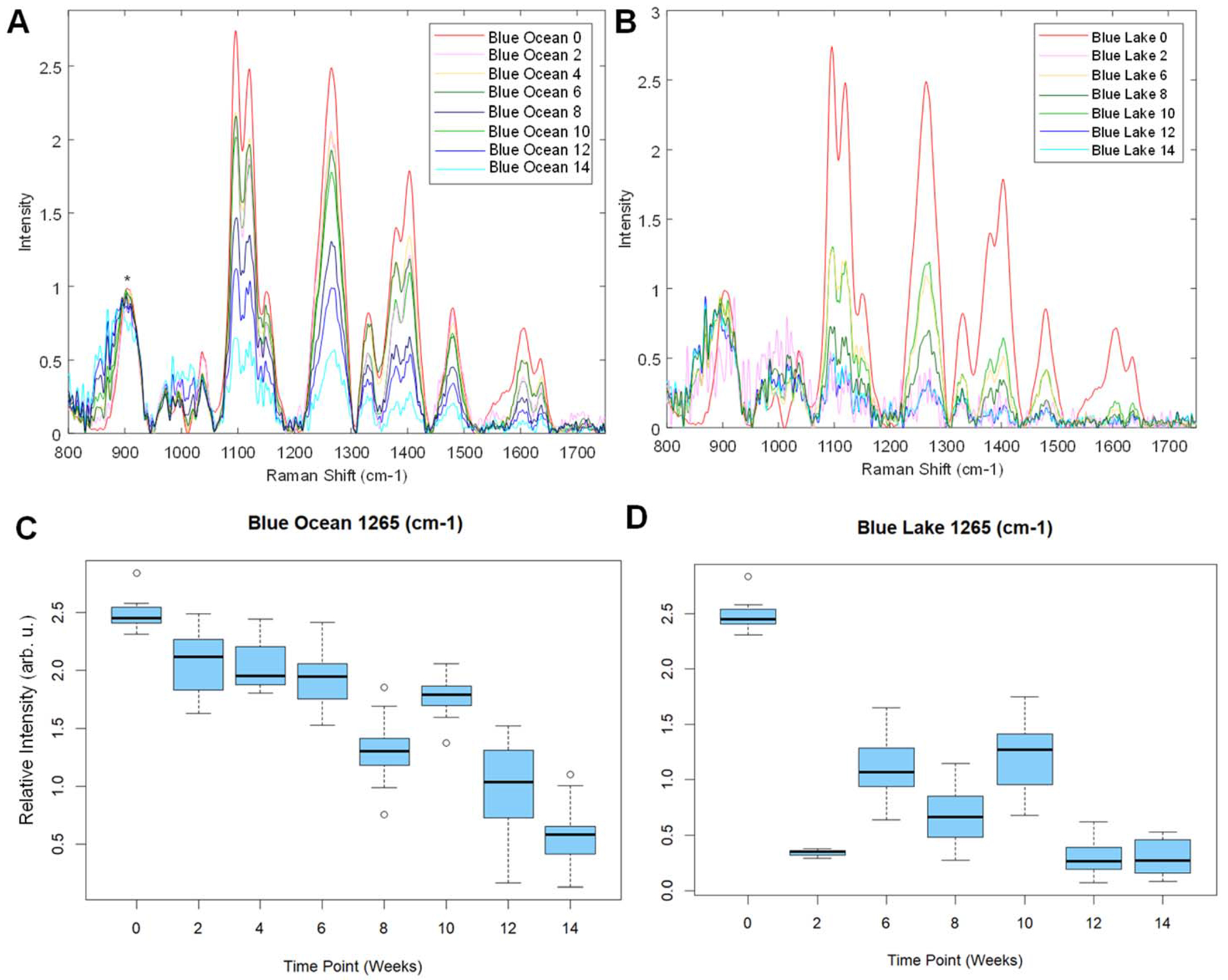
NIeRS spectra acquired from blue-colored cotton fabric exposed to ocean (A) and lake (B) waters. Corresponding changes in the 1265 cm^−1^ band are reflected in ANOVA graphs for the spectra acquired from ocean (C) and lake (D) water-exposed samples, as well as the control group (week 0).

NIeRS was also used to analyse spectroscopic changes observed in blue-coloured fabric exposed to the same environmental conditions, Fig. 4, Tables S3 and S4. We found that both ocean and lake waters triggered fading of the blue colorant. Similar to the discussed above results for magenta, we found that lake water triggered a much faster degradation of dyes compared to ocean water. Specifically, we observed nearly complete fading of the blue colorant already at week 2, while the same level of fading was observed for ocean waters by week 14.

**Fig. 5:**
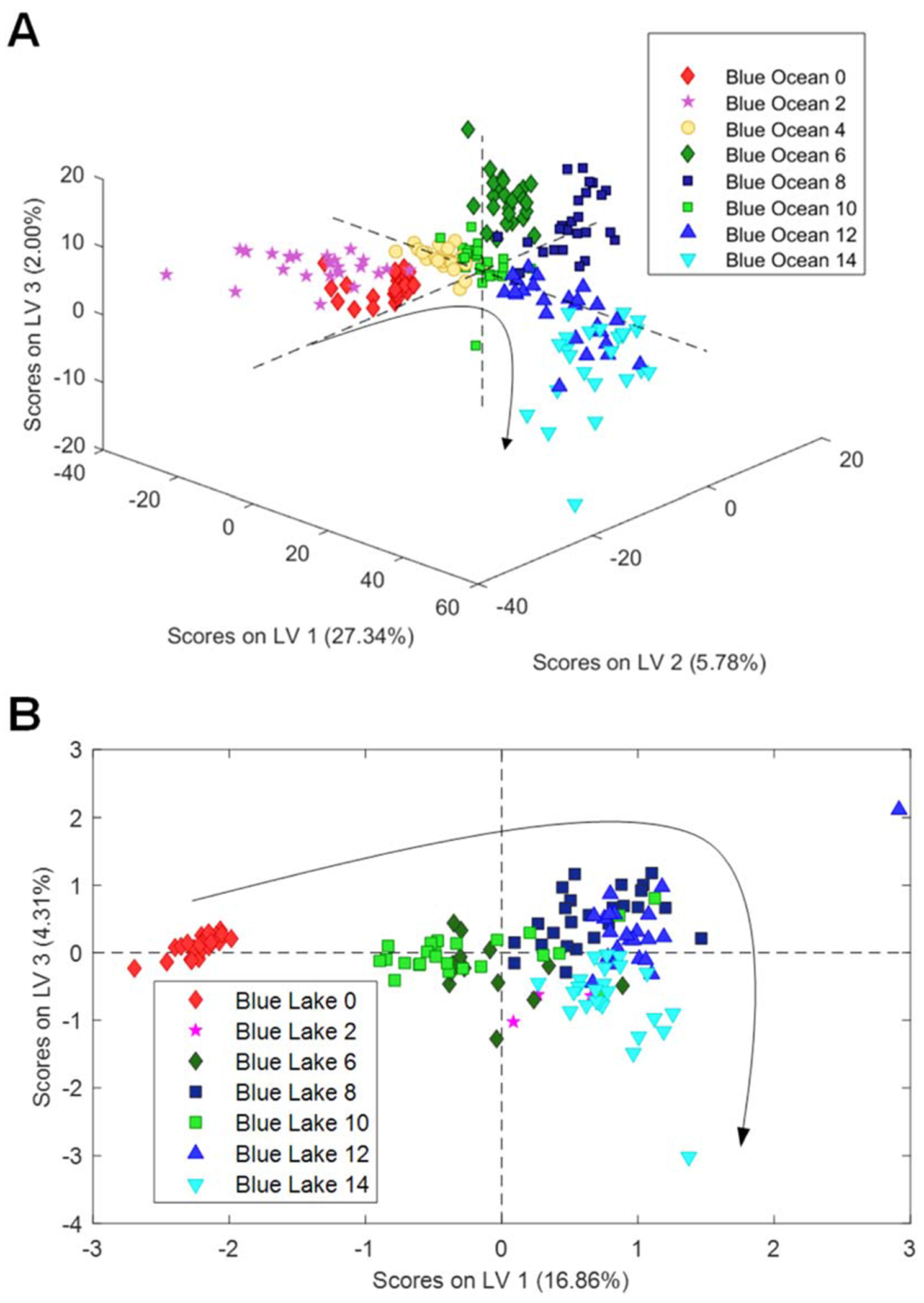
3D (A) and 2D (B) LV plots of NIeRS spectra acquired from blue-coloured fabric exposed to ocean (A) and lake (B) waters for 2–14 weeks, as well as the control group (week 0). Cross-Validation (CV) Error and Root Mean Square Error of Cross-Validation (RMSECV) Plots for these PLS-DA models are shown in Fig. S3 and S4.

The magnitude of dye fading is clearly visible in 3D and 2D plots, Fig. 5. Specifically, a gradient water-triggered fading was observed for ocean water conditions, while a drastic fading was observed for blue-coloured fabric exposed to lake water.

**Fig. 6:**
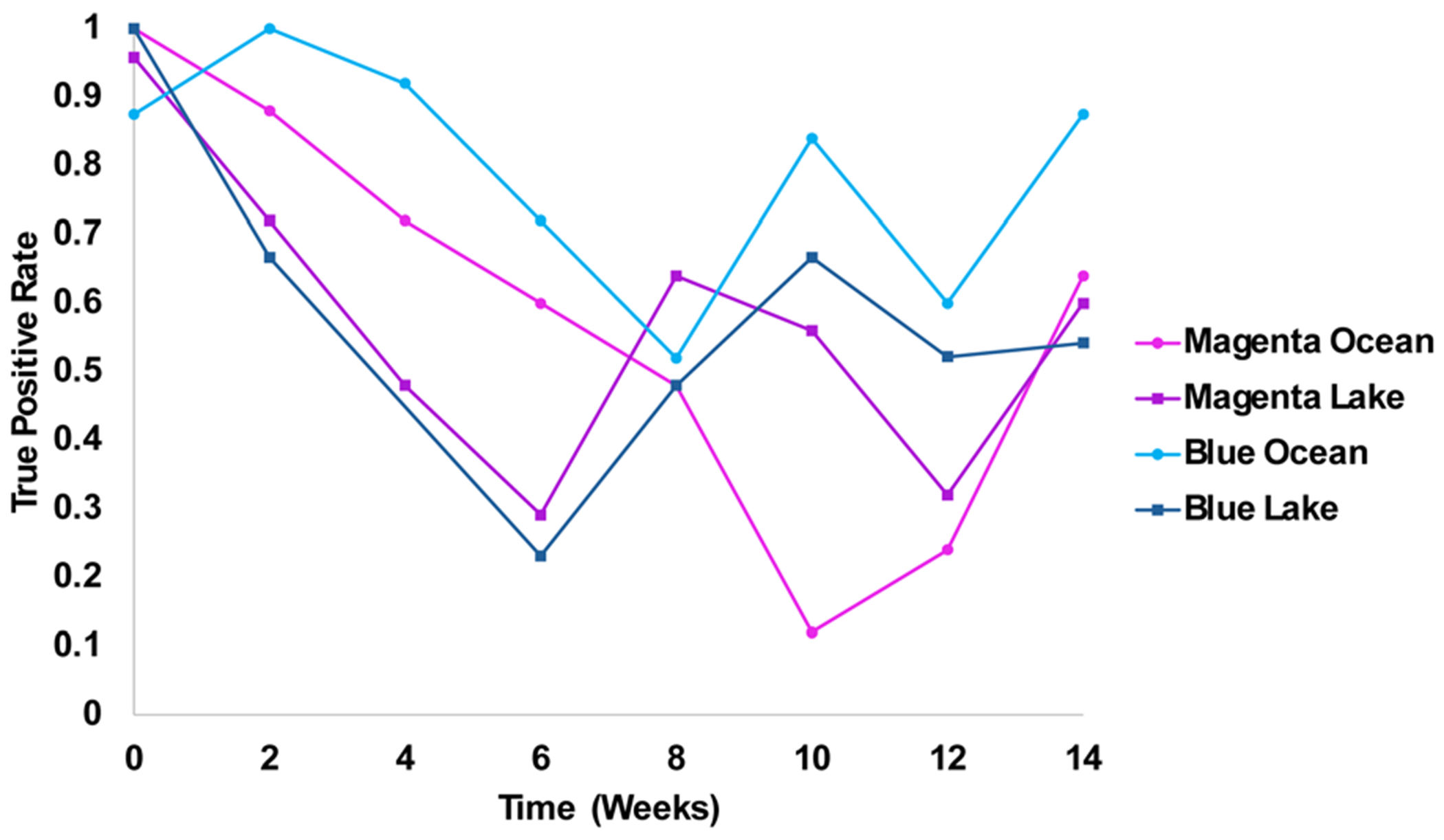
A graph of changes in true positive rate (TPR) of the identification of magenta and blue dyes on fabric exposed to ocean (A) and lake (B) waters for 2–14 weeks, as well as the control group (week 0).

Finally, we performed partial least-squared discriminant analysis (PLS-DA) to investigate the accuracy of NIeRS-based identification of two colorants exposed to both ocean and lake waters. We found that PLS-DA provide high (70–100%) accuracy within the first two weeks then lowered as the time of the exposure increased, Fig. 6. Next, we aimed to identify the dye present on fabric. To achieve this, we grouped all acquired spectra in two classes (blue and magenta) regardless of the time of the exposure, Table 2. Our results indicate that NIeRS coupled with chemometrics was able to identify blue and magenta dyes with 98.8% and 97.5% accuracies, respectively.

These results are consistent with previously reported findings from our group.^21^ Specifically, Bober and co-workers showed that sunlight exposure caused dye fading, which slightly lowered the accuracy of NIeRS-based identification of dyes on such fabric.^23^ Juarez and Kurouski found that PLS-DA coupled with NIeRS enabled 97.6% accurate identification of dyes on fabric contaminated with dry blood, urine and semen.^24^ It was also found that NIeRS could be used to identify blood, urine and semen on such fabric with 99.4% accuracy. Based on these results, we can conclude that NIeRS coupled with chemometrics allows for roust and reliable analysis of dyes on fabric exposed to various environmental conditions.

It should be noted that experimental conditions used in the current study only partially represent real forensic crime scenes. Specifically, fabric was kept in beakers with ocean and lake waters on the bench without direct sunlight exposure. Such exposure triggers dye fading,^23,25^ which would further shorten colorant detection window. We also think that constant washing with fresh water will minimize water–colorant equilibrium and accelerate dye fading. Finally, the rate of dye fading may depend on other environmental factors, including temperature, wildlife activity, etc. Therefore, future studies are required to fully understand the potential of NIeRS for detection and identification of dyes on fabric.

## Conclusions

In summary, our findings show that both ocean and lake water caused substantial fading of dyes on fabric. Our results indicate that lake water triggers stronger dye fading compared to ocean water. We hypothesized that this effect could be due to higher number of bacteria and microorganisms present in freshwater compared to salty ocean water. We also showed that NIeRS coupled with chemometrics could be used to identify the duration of fabric exposure to such environmental conditions. Finally, our results demonstrate that both magenta and blue dyes could be correctly predicted with ~98% accuracy. These results demonstrate that NIeRS is very efficient in the identification of dyes present on fabric exposed to water for the period up to 12 weeks.

## Experimental

### Colorants and dying procedures

A 100% cotton shirt purchased from fruit of the loom was used as the blank canvas for all samples. Blue and magenta dyes were purchased from Jacquard (https://store.jacquardproducts.com/). These dyes had reactive blue 4 and reactive red 2 colorants, respectively. Fabric was coloured following the manufacturer’s procedures. Briefly, dyes were dissolved in water and cotton fabric was submerged in the solution. Next, fabric was removed and dried under room temperature.

### Water exposure

Once fully dried, dyed cotton was submerged in water taken from the ocean (Galveston, TX) and lake (Lake Bryan, TX). Water was agitated using stirring bars for 14 weeks. Every two weeks, ~1 cm of fabric was cut off using scissors and dried under room temperature. To ensure complete submergence of remaining fabric, glass beakers that possessed fabric were refilled with corresponding waters biweekly. Photographs of fabric before exposure and after 14 weeks of exposure to ocean and lake water are shown in Fig. S6–S9.

### Spectral acquisition

To collect the NIeRS spectra, a handheld Agilent Resolve Spectrometer was used. The instrument is equipped with a 830 nm laser that was focused to a ~2 mm spot. Each spectrum was taken using a 1s acquisition time and 495 mW laser power. Spectra had an automatic baseline subtraction performed by the instrument. In total, ~25 spectra were taken from each sample. Scans were taken from different areas around the middle of each swatch for consistency between weeks.

### Spectral processing and statistical analysis

The spectral processing was made in PLS_Toolbox (Eigenvector Research, Inc., Manson, WA) integrated in Matlab (Mathworks). Spectra were smoothed by SavGol (order:2, window:15, polyinterp tails); MSC Median Normalization was used. PLS-DA models were trained with all spectra from the respective time points and cross-validation was used to predict accuracy of the model to predict the dyes. Classes were separated by the different time points. The number of latent variables (LV) used for each week’s model was chosen by the lowest point on its respective RMSECV 1 graph. ROC plots for PLS-DA models are shown in Fig. S10–S14. CV class. Error and/or RMSE values for each model are shown in Fig. S15. For intensity comparisons MSC Median Normalization was used and smoothed to the second polynomial (SavGol).^7^ ANOVA was used to analyse the fading of the dye over time.

## Supplementary Material

SI

Supplementary information (SI) is available. See DOI: https://doi.org/10.1039/d5ay01973g.

## Figures and Tables

**Table 1:** Vibrational bands in the NIeRS spectra acquired from magenta and blue dyes alone and coloured fabric

Sample	Vibrational bands, cm^−1^
Magenta	1218, 1279, 1336, 1359, 1425, 1490, and 1594
Magenta-on-cotton	903, 997, 1096, 1119, 1156, 1223, 1280, 1337, 1361, 1418, 1492, and 1594
Blue	910, 1041, 1128, 1162, 1266, 1327, 1369, 1404, 1487, 1609, and 1637
Blue-on-cotton	904, 970, 995, 1037, 1096, 1120, 1151, 1265, 1331, 1380, 1403, 1480, 1605, and 1635

**Table 2:** True positive (TPR) and false positive (FPR) rates of identification of blue and magenta dyes based on NIeRS spectra acquired from unexposed and exposed to waters samples. Cross-Validation (CV) Error and Root Mean Square Error of Cross-Validation (RMSECV) plots for this PLS-DA model are shown in Fig. S5

Predicted class	TPR %	FPR %	Blue	Magenta
Blue	98.8	2.52	332	10
Magenta	97.5	1.19	4	387

## Data Availability

Data used for this manuscript will be available upon request to the corresponding author.
