# Supplementary material for: Identification of dyes on fabric exposed to lake and ocean water using near-infrared excitation Raman spectroscopy
Source: Anal Methods. Author manuscript; Available in PMC 2026 Apr 6. (PMC13051311; doi:10.1039/d5ay01973g)
Supplement: SI [file NIHMS2157651-supplement-SI.docx]

Identification of Dyes on Fabric Exposed to Lake and Ocean Waters Using Near-Infrared Excitation Raman Spectroscopy

Claire Sasaki^1^, Shannon Bober^1^, Aidan P. Holman^1^ and Dmitry Kurouski^*1^

1. Department of Biochemistry and Biophysics, Texas A&M University, College Station, Texas 77843, United States

Supporting Information

 

Figure S1. Cross-Validation (CV) Error (left) and Root Mean Square Error of Cross-Validation (RMSECV) Plots (right) of PLS-DA model used to predict faring stages of magenta-colored cotton exposed to ocean water (Figure 3, A). Based on these plots, 4 latent variables (LVs) were chosen.


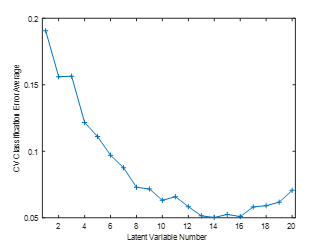

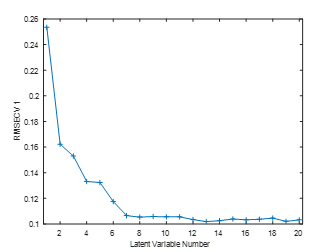


Figure S2. Cross-Validation (CV) Error (left) and Root Mean Square Error of Cross-Validation (RMSECV) Plots (right) of PLS-DA model used to predict faring stages of magenta-colored cotton exposed to lake water (Figure 3, B). Based on these plots, 2 latent variables (LVs) were chosen.

Figure S3. Cross-Validation (CV) Error (left) and Root Mean Square Error of Cross-Validation (RMSECV) Plots (right) of PLS-DA model used to predict faring stages of blue-colored cotton exposed to ocean water (Figure 5, A). Based on these plots, 5 latent variables (LVs) were chosen.


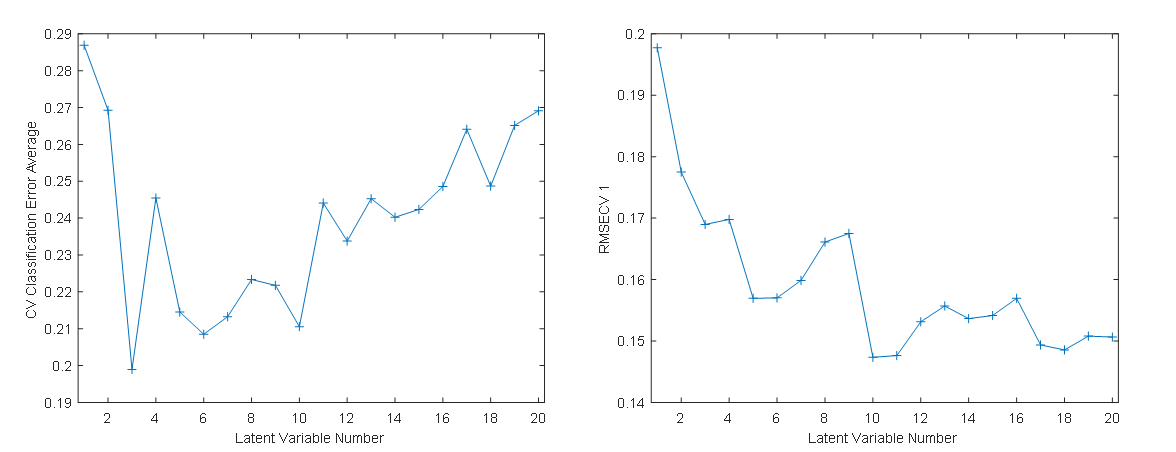


Figure S4. Cross-Validation (CV) Error (left) and Root Mean Square Error of Cross-Validation (RMSECV) Plots (right) of PLS-DA model used to predict faring stages of blue-colored cotton exposed to ocean lake (Figure 5, B). Based on these plots, 5 latent variables (LVs) were chosen.

Figure S5. Cross-Validation (CV) Error (left) and Root Mean Square Error of Cross-Validation (RMSECV) Plots (right) of PLS-DA model used to predict dyes on colored cotton exposed to ocean and lake water (Table 2). Based on these plots, 6 latent variables (LVs) were chosen.


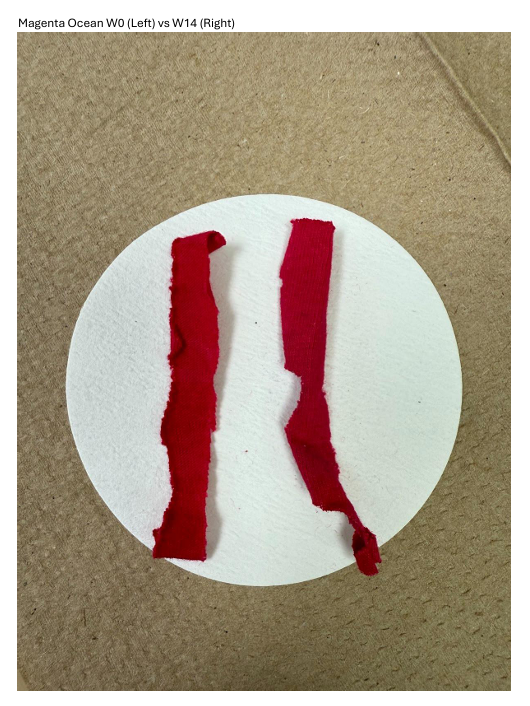


Figure S6. Magenta-colored fabric at week 0 (left) and week 14 (right) after exposure to ocean water.


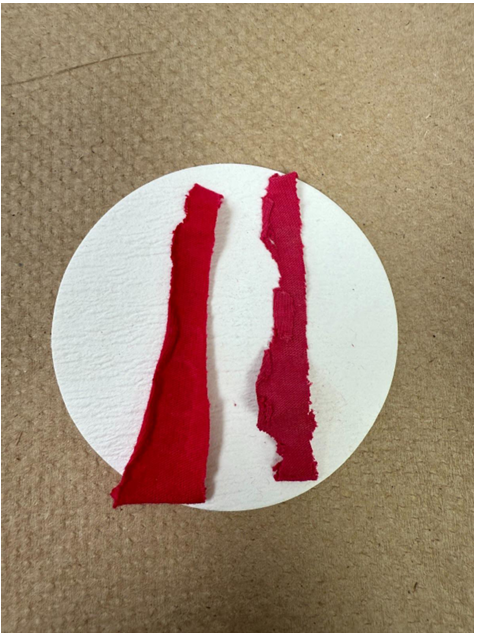


Figure S7. Magenta-colored fabric at week 0 (left) and week 14 (right) after exposure to lake water.


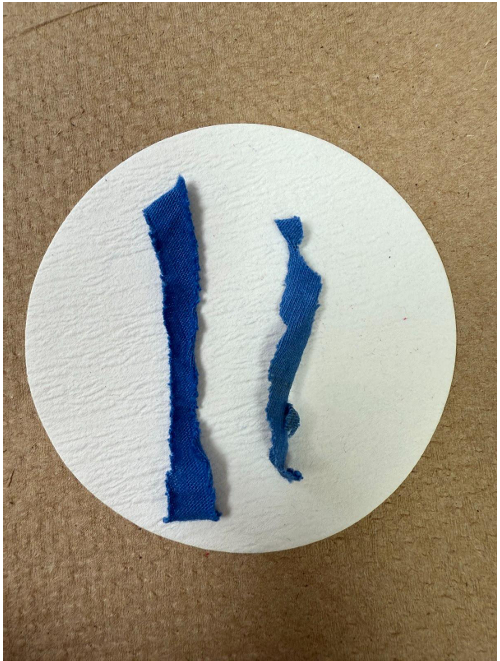


Figure S8. Blue-colored fabric at week 0 (left) and week 14 (right) after exposure to ocean water.


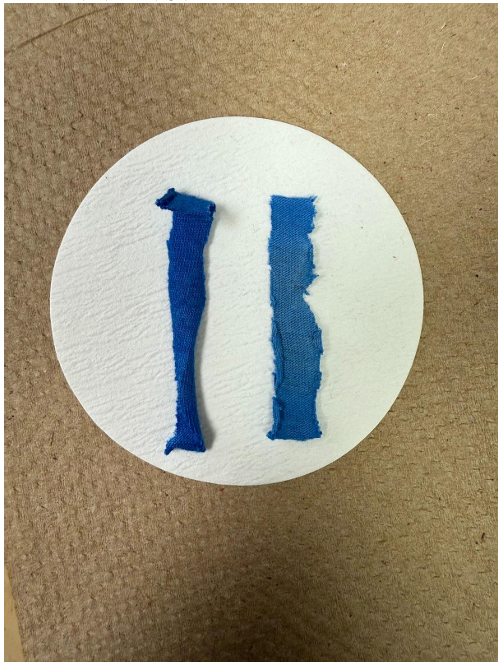


Figure S9. Blue-colored fabric at week 0 (left) and week 14 (right) after exposure to lake water.

**Figure S10.** ROC plots for PLS-DA model used to predict faring stages of magenta-colored cotton exposed to ocean water (Figure 3, A).

**Figure S11.** ROC plots for PLS-DA model used to predict faring stages of magenta-colored cotton exposed to lake water (Figure 3, A).

**Figure S12.** ROC plots for PLS-DA model used to predict faring stages of blue-colored cotton exposed to ocean water (Figure 5, A).

**Figure S13.** ROC plots for PLS-DA model used to predict faring stages of blue-colored cotton exposed to lake water (Figure 5, A).

**Figure S14.** ROC plots for PLS-DA model used to predict faring stages of blue-colored cotton exposed to ocean and lake water (Table 2).

**Figure S15.** CV classification error and RMSE values for each model.

**Table S1.** P-values for ANOVA of magenta-colored fabric exposed to ocean water.

| Comparison | p |
| --- | --- |
| 0-10 | < 0.001 |
| 0-12 | < 0.001 |
| 0-14 | < 0.001 |
| 0-4 | 0.006 |
| 0-6 | < 0.001 |
| 0-8 | < 0.001 |
| 10-12 | 0.005 |
| 10-14 | < 0.001 |
| 10-2 | < 0.001 |
| 12-2 | < 0.001 |
| 12-4 | < 0.001 |
| 12-8 | 0.049 |
| 14-2 | < 0.001 |
| 14-4 | < 0.001 |
| 14-8 | 0.012 |
| 2-4 | 0.034 |
| 2-6 | < 0.001 |
| 2-8 | < 0.001 |
| 4-6 | 0.002 |
| 4-8 | .013 |

**Table S2.** P-values for ANOVA of magenta-colored fabric exposed to lake water.

| Comparison | p |
| --- | --- |
| 0-10 | < 0.001 |
| 0-12 | < 0.001 |
| 0-14 | < 0.001 |
| 0-4 | 0.015 |
| 0-6 | < 0.001 |
| 0-8 | < 0.001 |
| 10-12 | < 0.001 |
| 10-14 | < 0.001 |
| 10-2 | 0.012 |
| 10-6 | 0.042 |
| 10-8 | 0.007 |
| 12-2 | < 0.001 |
| 12-4 | < 0.001 |
| 12-6 | 0.006 |
| 12-8 | 0.030 |
| 14-2 | < 0.001 |
| 14-4 | < 0.001 |
| 14-6 | < 0.001 |
| 14-8 | < 0.001 |
| 2-6 | < 0.001 |
| 2-8 | < 0.001 |
| 4-6 | 0.001 |
| 4-8 | < 0.001 |

**Table S3.** P-values for ANOVA of blue-colored fabric exposed to ocean water.

| Comparison | p |
| --- | --- |
| 0-10 | < 0.001 |
| 0-12 | < 0.001 |
| 0-14 | < 0.001 |
| 0-2 | 0.004 |
| 0-4 | 0.003 |
| 0-6 | < 0.001 |
| 0-8 | < 0.001 |
| 10-12 | < 0.001 |
| 10-14 | < 0.001 |
| 10-2 | 0.024 |
| 10-4 | 0.031 |
| 10-8 | 0.008 |
| 12-2 | < 0.001 |
| 12-4 | < 0.001 |
| 12-6 | < 0.001 |
| 14-2 | < 0.001 |
| 14-4 | < 0.001 |
| 14-6 | < 0.001 |
| 14-8 | 0.013 |
| 2-8 | < 0.001 |
| 4-8 | < 0.001 |
| 6-8 | < 0.001 |

**Table S4.** P-values for ANOVA of blue-colored fabric exposed to lake water.

| Comparison | p |
| --- | --- |
| 0-10 | 0.006 |
| 0-12 | < 0.001 |
| 0-14 | < 0.001 |
| 0-2 | < 0.001 |
| 0-6 | 0.007 |
| 0-8 | < 0.001 |
| 10-12 | < 0.001 |
| 10-14 | < 0.001 |
| 10-2 | 0.015 |
| 10-8 | 0.006 |
| 12-6 | < 0.001 |
| 12-8 | 0.002 |
| 14-6 | < 0.001 |
| 14-8 | 0.002 |
| 2-6 | 0.034 |
